# Deep learning radiomics of dual-modality ultrasound images for hierarchical diagnosis of unexplained cervical lymphadenopathy

**DOI:** 10.1186/s12916-022-02469-z

**Published:** 2022-08-26

**Authors:** Yangyang Zhu, Zheling Meng, Xiao Fan, Yin Duan, Yingying Jia, Tiantian Dong, Yanfang Wang, Juan Song, Jie Tian, Kun Wang, Fang Nie

**Affiliations:** 1 Ultrasound Medical Center, Lanzhou University Second Hospital, Lanzhou University, Lanzhou, 730030 China; 2 CAS Key Laboratory of Molecular Imaging, The State Key Laboratory of Management and Control for Complex Systems, Institute of Automation, Chinese Academy of Sciences, Beijing, 100190 China; 3 School of Artificial Intelligence, University of Chinese Academy of Sciences, Beijing, China; 4 Department of Ultrasound, Gansu Provincial Cancer Hospital, Lanzhou, China; 5 Department of Ultrasound, People’s Hospital of Ningxia Hui Autonomous Region, Yinchuan, China; 6 Beijing Advanced Innovation Center for Big Data-Based Precision Medicine, School of Medicine and Engineering, Beihang University, Beijing, China; 7 Gansu Province Clinical Research Center for Ultrasonography, Lanzhou, China; 8 Gansu Province Medical Engineering Research Center for Intelligence Ultrasound, Lanzhou, China

**Keywords:** Deep learning, Cervical lymphadenopathy, Ultrasound, Reactive hyperplasia, Tuberculous lymphadenitis, Lymphoma, Metastatic carcinoma

## Abstract

**Background:**

Accurate diagnosis of unexplained cervical lymphadenopathy (CLA) using medical images heavily relies on the experience of radiologists, which is even worse for CLA patients in underdeveloped countries and regions, because of lack of expertise and reliable medical history. This study aimed to develop a deep learning (DL) radiomics model based on B-mode and color Doppler ultrasound images for assisting radiologists to improve their diagnoses of the etiology of unexplained CLA.

**Methods:**

Patients with unexplained CLA who received ultrasound examinations from three hospitals located in underdeveloped areas of China were retrospectively enrolled. They were all pathologically confirmed with reactive hyperplasia, tuberculous lymphadenitis, lymphoma, or metastatic carcinoma. By mimicking the diagnosis logic of radiologists, three DL sub-models were developed to achieve the primary diagnosis of benign and malignant, the secondary diagnosis of reactive hyperplasia and tuberculous lymphadenitis in benign candidates, and of lymphoma and metastatic carcinoma in malignant candidates, respectively. Then, a CLA hierarchical diagnostic model (CLA-HDM) integrating all sub-models was proposed to classify the specific etiology of each unexplained CLA. The assistant effectiveness of CLA-HDM was assessed by comparing six radiologists between without and with using the DL-based classification and heatmap guidance.

**Results:**

A total of 763 patients with unexplained CLA were enrolled and were split into the training cohort (*n*=395), internal testing cohort (*n*=171), and external testing cohorts 1 (*n*=105) and 2 (*n*=92). The CLA-HDM for diagnosing four common etiologies of unexplained CLA achieved AUCs of 0.873 (95% CI: 0.838–0.908), 0.837 (95% CI: 0.789–0.889), and 0.840 (95% CI: 0.789–0.898) in the three testing cohorts, respectively, which was systematically more accurate than all the participating radiologists. With its assistance, the accuracy, sensitivity, and specificity of six radiologists with different levels of experience were generally improved, reducing the false-negative rate of 2.2–10% and the false-positive rate of 0.7–3.1%.

**Conclusions:**

Multi-cohort testing demonstrated our DL model integrating dual-modality ultrasound images achieved accurate diagnosis of unexplained CLA. With its assistance, the gap between radiologists with different levels of experience was narrowed, which is potentially of great significance for benefiting CLA patients in underdeveloped countries and regions worldwide.

**Supplementary Information:**

The online version contains supplementary material available at 10.1186/s12916-022-02469-z.

## Background

Cervical lymphadenopathy (CLA) is a common disease occurring in patients of all ages, with an annual incidence of 0.6–0.7% for the general population [1, 2]. The most common etiologies are reactive hyperplasia (38–79%) and tuberculous lymphadenitis (4–34%) in benign cases and metastatic carcinoma (50–94%) and lymphomas (5–41%) in malignant cases [3–6]. Referral patterns and treatment strategies for different types of CLA are all distinct; thus, accurate identification of the specific etiology is essential for subsequent medical management [1, 7]. However, the differential diagnosis of CLA is challenging, especially in patients without reliable medical history and characteristic symptoms, which is commonly seen in underdeveloped areas of developing countries [8]. Due to the lack of a universally accepted protocol for the investigation of lymphadenopathy, some of these unexplained CLA may experience an average delay of 3 to 6 months from the initial presentation of symptoms to the diagnosis of malignancy [9]. Recently, specialized lymph node diagnostic clinics have been established in several developed countries and advanced medical institutions to benefit unexplained CLA patients with rapid, agile, and scheduled systems, but same interventions are still impractical in many countries and regions with underdeveloped healthcare conditions, involving a huge population worldwide [1, 10, 11].

Imaging methods are the main tools for detection, diagnosis, and follow-up monitoring in unexplained CLA patients, including ultrasound imaging (US), computed tomography (CT), and magnetic resonance imaging (MRI). Compared with other imaging modalities, US is more convenient, economical, and radiation-free and has better resolution in characterizing cervical lymph nodes (CLNs). It consists of two basic modalities, B-mode ultrasound (BUS) and color Doppler flow imaging (CDFI), where BUS reliably shows the size, shape, borders, and internal echoes of the CLN, while CDFI is utilized to complement BUS by detecting blood vessels and assessing the vascular distribution of the CLN in real time [12]. The importance of BUS and CDFI duplex ultrasound in patients with CLA is well recognized, and this method is recommended as the first-line diagnostic tool for unexplained CLA [11, 13]. However, the diagnostic performance of the dual-modality US strongly relies on the clinical and professional expertise of radiologists [14, 15]. Subjective image interpretation, lack of effective quantification, and persistent intra- and inter-observer variability remain the main dilemmas faced in US examinations. Consequently, a significant proportion of patients with unexplained CLA are frequently misdiagnosed and subsequently subjected to unnecessary investigations and inappropriate treatment [16].

To enable timely and accurate diagnosis of unexplained CLA patients with relatively less demand for clinical expertise, one potentially promising approach is utilizing artificial intelligence (AI) technology. AI technology represented by radiomics can mine high-throughput quantitative features from image data to reveal disease features and includes two main strategies of machine learning and deep learning (DL). Some inherent characteristics of ultrasound images (including limited image quality and susceptibility to operator influence) can make the manual definition and extraction of image features less reliable, limiting the performance of traditional machine learning. Meanwhile, DL has gradually started to become a mainstream research method for ultrasound image analysis by using deep neural networks and data-driven learning techniques to achieve automatic extraction and quantification of image features imperceptible by naked eyes [17–19]. Recent studies have shown that DL has achieved good performance in diagnosing thyroid nodules [20], classifying parotid gland tumors [21], identifying extra-nodal extension of head and neck squamous cell carcinoma [22], predicting prognosis of oral cancer [23], and detecting COVID-19 pneumonia [24]. However, its application in the context of lymph node imaging is still rare, and only few studies reported that DL with BUS images of lymph nodes could identify whether relevant draining lymph nodes of breast [25], thyroid [26], and lung cancer [27] were metastatic or not. To the best of our knowledge, it has not been used in the characterization of unexplained CLA yet.

In this study, we developed a cervical lymphadenopathy hierarchical diagnosis model (CLA-HDM) based on DL radiomics. It used BUS and CDFI dual-modality images to establish a two-level diagnostic structure for unexplained CLA. CLA-HDM mimics the clinical diagnosis logic and divides the characterization task into three sub-tasks. It firstly classifies unexplained CLA as benign or malignant and then determined the specific etiology in each condition. It was trained and validated (both internally and externally) in multi-center unexplained CLA patient cohorts. The performances of different radiologists were compared between with and without CLA-HDM’s assistance. The model was opened for external validation.

## Methods

### Study cohorts

This multi-center diagnostic study, conducted from June 1, 2018, and November 31, 2021, was approved by the ethics committee of the Second Hospital of Lanzhou University, and the requirement for individual consent for this retrospective analysis was waived. This study followed the Standards for Reporting of Diagnostic Accuracy guidelines.

**Fig. 1:**
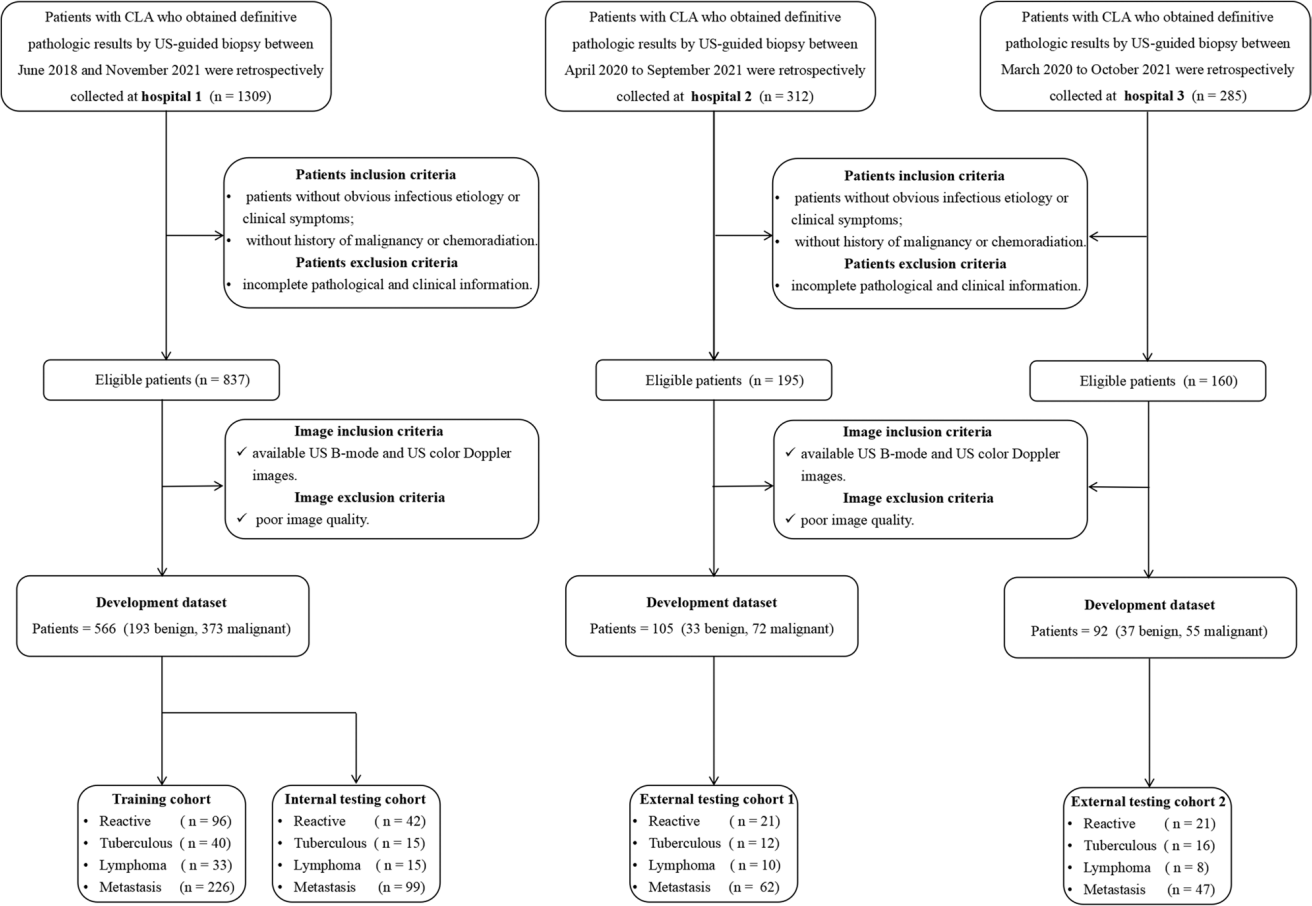
Patient selection flowchart. CLA, cervical lymphadenopathy; US, ultrasound

1906 patients were collected from three hospitals located in underdeveloped areas of China (hospital 1: Lanzhou University Second Hospital; hospital 2: Gansu Provincial Cancer Hospital; hospital 3: People’s Hospital of Ningxia Hui Autonomous Region), who all had definitive CLA pathological findings by US-guided needle and/or excisional biopsy. Excision biopsy was required only when the needle biopsy result was inconclusive. The following inclusion criteria were applied: (a) patients without obvious infectious etiology or clinical symptoms (e.g., tenderness and fever), (b) patients without history of malignancy or chemoradiation, and (c) patients with available BUS and CDFI images. The exclusion criteria were as follows: (a) patients with incomplete pathological and clinical information and (b) patients with poor BUS or CDFI images. The flowchart of the patient inclusion criterion is shown in Fig. 1.

### Image acquisition

All examinations of patients (ultrasound and LN biopsy) at the three hospitals involved were performed by radiologists with more than 10 years of ultrasound experience, and the ultrasound images of these patients were obtained from 14 different diagnostic ultrasound instruments (the process of ultrasound images collection is shown in Additional file 1: Methods 1; details of the instruments used in each hospital can be found in Additional file 1: Table S1). In accordance with the clinical practice, the selected lymph node for biopsy at each hospital was the most suspicious lymph node on images (the largest suspicious lymph node was selected when multiple suspicious lymph nodes were present) [12, 28]. Baseline characteristics (sex, age, node longitudinal diameter, location, neck level, and methods of pathologic diagnosis) of the patients and selected lymph nodes were obtained from electronic medical records and biopsy reports.

### Model development

**Fig. 2:**
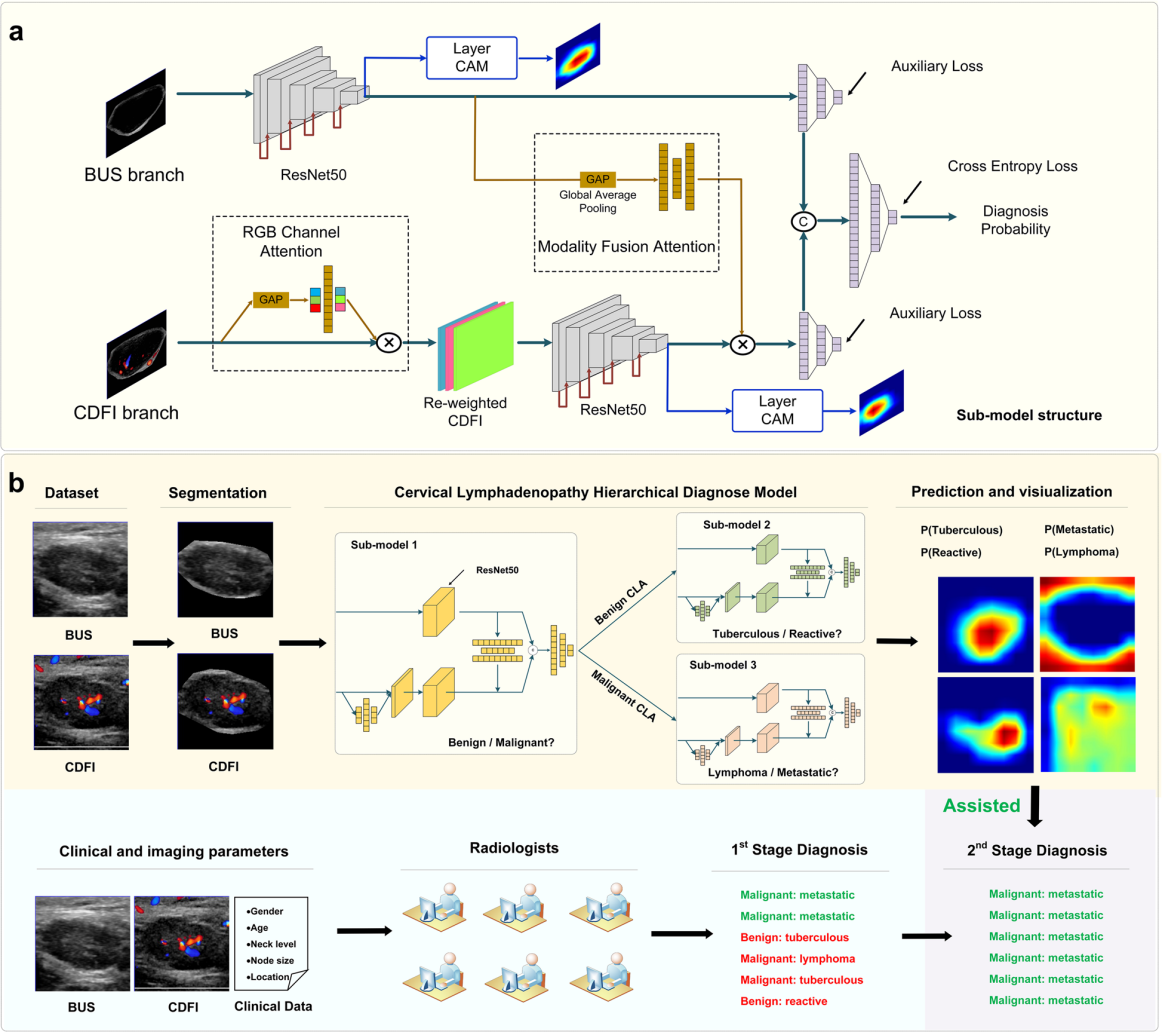
Proposed deep learning-based hierarchical diagnostic model (CLA-HDM) to non-invasively assess unexplained CLA. **a** Each sub-model takes BUS and CDFI images as inputs and assigns weights between different color channels in CDFI branch and pays attention to specific CDFI features under the guidance of BUS branch via attention mechanism. **b** For each test case, our model utilizes dual-modal ultrasound images as inputs each time, outputs hierarchical diagnostic task-related predictive probabilities and corresponding heatmaps to compare with and assist radiologists. CLA, cervical lymphadenopathy; BUS, B-mode ultrasound; CDFI, color Doppler flow imaging; AI, artificial intelligence

CLA-HDM consisted of three task-specific classification sub-models, including sub-model 1 for the diagnosis of benign and malignant unexplained CLA, sub-model 2 for the diagnosis of tuberculous and reactive in the set of benign type candidates, and sub-model 3 for the diagnosis of metastatic and lymphoma in the set of malignant type candidates (Fig. 2). Each sub-model had a dual branch and late-fusion structure with two attention blocks. The two branches took BUS and CDFI images as inputs respectively. A fully connected layer like channel attention block was applied to reweight the R, G, and B channels to highlight the important color information in CDFI. Then, the BUS and channel-reweighted CDFI images were fed forward into their respective feature extractors (ResNet-50 [29]). Modality fusion attention was applied to the features in the CDFI branch, and its weights were obtained from the features of the BUS branch by global average pooling and fully connected layer, in order to mimic radiologists who read CDFI images primarily based on the understanding of corresponding BUS images. These three task-specific sub-models shared the same structure but not parameters (Fig. 2a).

In the training stage, we trained three sub-models independently on the training cohort. In the testing stage, we firstly evaluated the performance of the sub-models individually. Then, the three sub-models were assembled to build CLA-HDM to diagnose every case in the testing cohorts. Whether CLA-HDM would output the diagnosis probability of sub-model 2 or sub-model 3 was automatically determined by the diagnosis result of sub-model 1(Additional file 1: Method S2). Layer-CAM [30, 31] was applied to the final stage feature maps of the feature extractors to visualize the heatmaps (Additional file 1: Method S5). Details of the methods, including data preprocessing and model development, are shown in Additional file 1: Method S1, S3 and S4 [32–41].

### Radiologist study

A two-stage radiologist study was conducted to evaluate the diagnostic performance of the CLA-HDM and its clinical application value. Six radiologists with an average of 10 years of US experience (3–20 years) participated in this study, and they were divided into three groups according to the years of experience: seniors (radiologist 1 [F.N.], 20 years; radiologist 2 [Y.D.], 14 years), middles (radiologist 3 [Y.Y.J.], 9 years; radiologist 4 [T.T.D.], 8 years), and juniors (radiologist 5 [Y.F.W.], 5 years; radiologist 6 [X.F.], 3 years). The testing cohorts were shuffled and submitted to radiologists. Each radiologist was asked to interpret them blindly and independently.

In the first stage of radiologist study, the BUS images, CDFI images, and baseline characteristics of each patient were available for radiologists. Each radiologist first classifies unexplained CLA as benign or malignant, and then they further determined specific etiology. In the second stage (AI-assisted radiologist study), the corresponding lymph node hierarchical diagnostic heatmaps and AI probabilities were provided for the radiologists. Each radiologist was allowed to change or maintain the initial diagnosis and gave the final diagnosis conclusions (Fig. 2b).

### Statistical analysis

All statistical analyses were performed using SPSS software (version 26.0) and Python (version 3.8.10). Continuous variables were expressed as means ± standard deviations, and comparisons between two groups were made using the Mann-Whitney *U* test or Student’s *t*-test. Categorical variables were expressed as numbers and percentages, and comparisons between two groups were made using the chi-squared test or Fisher’s exact test. ROC analysis was used to evaluate the diagnostic performance of the model in the training and testing cohorts (micro-averaging was used to plot multi-class ROC [42]). 95% confidence interval (CI) was calculated using bootstrapping with 1000 resamples. Differences in performance between CLA-HDM and six radiologists and among six individual radiologists without and with AI assistance were assessed using McNemar’s test. Diagnostic performance between the CLA-HDM and three different levels of radiologist groups and between different radiologist groups was compared using a permutation test. Statistical significance was set at *P* < 0.05.

## Results

A total of 763 unexplained CLA patients were successfully enrolled in this multi-center study (Fig. 1), and the detailed pathological diagnostic results are shown in Additional file 1: Table S2. Of these, 566 cases from hospital 1 were used as the primary cohort to reduce overfitting or bias in the analysis. Cases before 2021 were selected in the primary cohort as the training cohort (*n* = 395) for model development, while cases from 2021 were used as the internal testing cohort (*n* =171) to simulate prospective experimental conditions. Cases from hospitals 2 (*n* = 105) and 3 (*n* = 92) were used as external test cohorts 1 and 2, respectively. There were no clinically significant differences between the training and three testing cohorts (*P* > 0.05; Additional file 1: Table S3), and all testing cohorts were used for radiologist-machine comparison.

### Sub-model performance evaluation

**Fig. 3:**
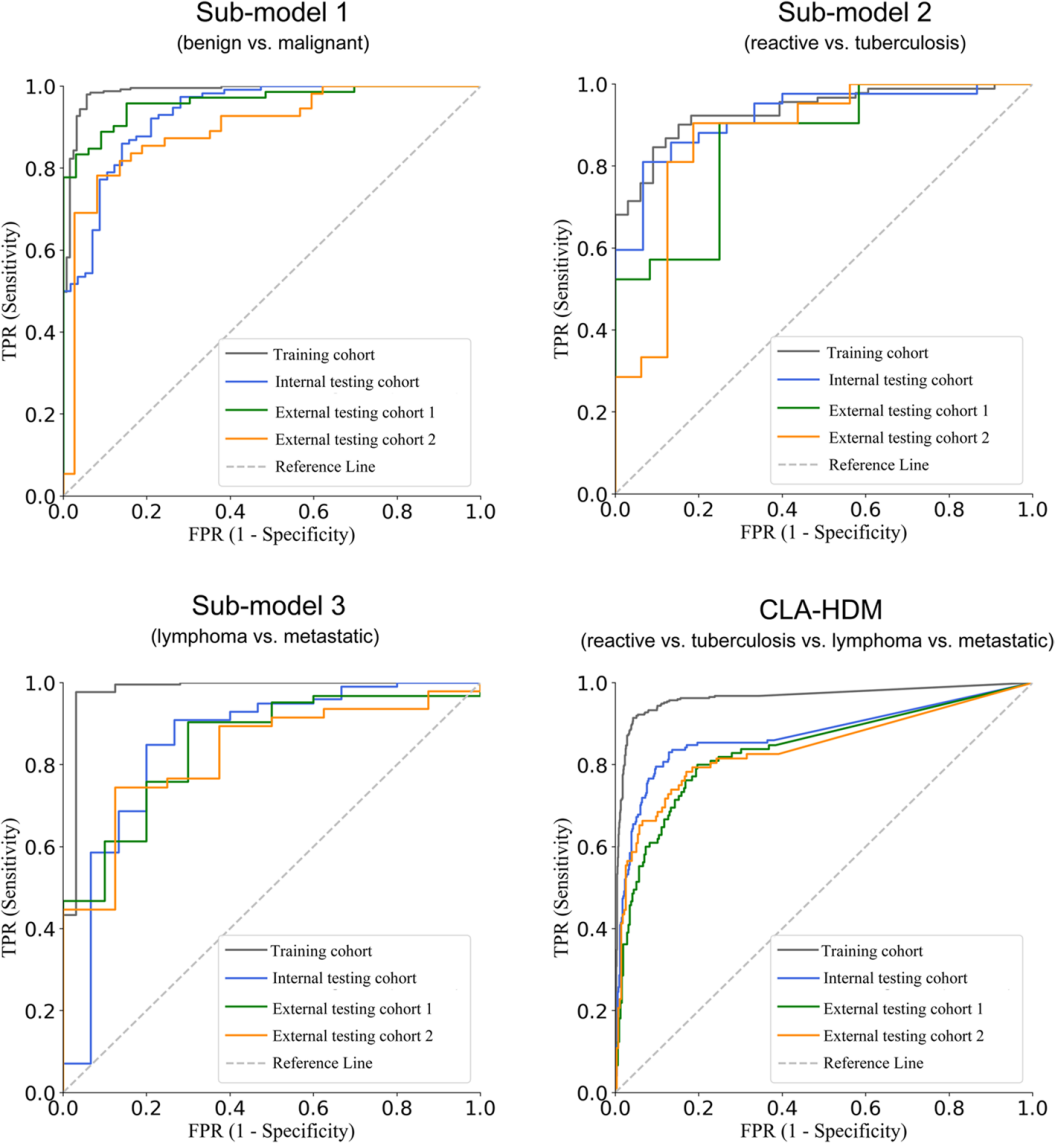
Diagnostic performance of three task-specific sub-models and their assembled model (CLA-HDM) in the training cohort, internal testing cohort, and external testing cohort 1 and 2

The performance of three sub-models was tested independently. In the internal testing, and external testing cohorts 1 and 2, sub-model 1 showed AUCs of 0.932, 0.963, and 0.896; an accuracy of 86.0%, 87.6%, and 82.6%; a sensitivity of 89.5%, 83.3%, and 81.8%; and a specificity of 78.9%, 96.9%, and 83.8% for differentiation between benign and malignant unexplained CLA. Sub-model 2 showed AUCs of 0.922, 0.857, and 0.872; an accuracy of 84.2%, 75.8%, and 78.4%; a sensitivity of 85.7%, 76.2%, and 71.4%; and a specificity of 80.0%, 75.0%, and 87.5% for differentiation between tuberculous lymphadenitis and reactive hyperplasia. Sub-model 3 showed AUCs of 0.852, 0.847, and 0.827; an accuracy of 86.0%, 86.1%, and 83.6%; a sensitivity of 87.9%, 88.7%, and 87.2%; and a specificity of 73.3%, 70.0%, and 62.5% for differentiation between lymphoma and metastatic carcinoma (Table 1 and Fig. 3).

**Table 1:** Performance of sub-models and CLA-HDM in the diagnosis of unexplained CLA

Models	Cohorts	AUC	ACC (%)	SENS (%)	SPEC (%)
Sub-model 1	Training cohort (*n* = 395)	0.986 (0.977, 0.998)	96.8 (95.2, 98.4)	97.9 (96.7, 99.7)	94.4 (91.1, 98.0)
	Internal testing cohort (*n* = 171)	0.932 (0.901, 0.966)	86.0 (81.9, 90.1)	89.5 (85.2, 94.5)	78.9 (70.2, 88.3)
	External testing cohort 1 (*n* = 105)	0.963 (0.939, 0.993)	87.6 (82.9, 93.3)	83.3 (76.7, 90.9)	96.9 (93.9, 103.0)
	External testing cohort 2 (*n* = 92)	0.896 (0.846, 0.963)	82.6 (76.1, 90.2)	81.8 (73.4, 90.9)	83.8 (74.0, 95.2)
Sub-model 2	Training cohort (*n* = 136)	0.935 (0.902, 0.976)	86.3 (81.5, 91.9)	84.6 (78.7, 91.1)	90.9 (81.8, 100.0)
	Internal testing cohort (*n* = 57)	0.922 (0.866, 0.986)	84.2 (77.2, 91.2)	85.7 (76.8, 94.8)	80.0 (65.6, 97.5)
	External testing cohort 1 (*n* = 33)	0.857 (0.758, 0.981)	75.8 (63.6, 87.9)	76.2 (61.9, 93.6)	75.0 (56.7, 96.2)
	External testing cohort 2 (*n* = 37)	0.872 (0.771, 0.986)	78.4 (67.6, 89.2)	71.4 (54.9, 87.9)	87.5 (75.0, 102.8)
Sub-model 3	Training cohort (*n* = 259)	0.979 (0.96, 1.012)	93.2 (90.8, 96.0)	92.6 (89.9, 95.6)	96.9 (93.8, 102.6)
	Internal testing cohort (*n* = 114)	0.852 (0.759, 0.968)	86.0 (80.7, 91.2)	87.9 (82.8, 93.4)	73.3 (55.0, 93.3)
	External testing cohort 1 (*n* = 72)	0.847 (0.742, 0.969)	86.1 (79.2, 93.1)	88.7 (82.2, 95.8)	70.0 (40.0, 97.1)
	External testing cohort 2 (*n* = 55)	0.827 (0.715, 0.964)	83.6 (76.4, 92.7)	87.2 (78.9, 95.6)	62.5 (35.0, 91.7)
CLA-HDM	Training cohort (*n* = 395)	0.964 (0.951, 0.978)	94.1 (92.6, 95.6)	88.2 (85.3, 91.2)	96.1 (95.1, 97.1)
	Internal testing cohort (*n* = 171)	0.873 (0.838, 0.908)	87.1 (84.2, 90.1)	74.3 (68.4, 80.1)	91.4 (89.5, 93.4)
	External testing cohort 1 (*n* = 105)	0.837 (0.789, 0.889)	82.9 (78.6, 86.7)	65.7 (57.1, 73.3)	88.6 (85.7, 91.1)
	External testing cohort 2 (*n* = 92)	0.840 (0.789, 0.898)	85.9 (82.1, 89.7)	71.7 (64.1, 79.3)	90.6 (88.0, 93.1)

The data in brackets represent the 95% confidence intervals

### CLA-HDM performance evaluation

After integrating three sub-models together, CLA-HDM designed for diagnosing four common etiologies of unexplained CLA (reactive, tuberculosis, lymphoma, and metastatic) achieved the overall AUCs of 0.873 (95% CI, 0.838–0.908), 0.837 (95% CI, 0.789–0.889), and 0.840 (95% CI, 0.789–0.898) in three testing cohorts, respectively (Table 1 and Fig. 3). More specifically, AUCs for reactive hyperplasia were 0.718 (95% CI, 0.595–0.856), 0.875 (95% CI, 0.793–0.967), and 0.812 (95% CI, 0.691–0.952); for tuberculous lymphadenitis were 0.883 (95% CI, 0.830–0.939), 0.860 (95% CI, 0.795–0.938), and 0.897 (95% CI, 0.828–0.976); for lymphoma were 0.816 (95% CI, 0.685–0.964), 0.670 (95% CI, 0.518–0.843), and 0.936 (95% CI, 0.884–1.006); and for metastatic carcinoma were 0.855 (95% CI, 0.811–0.906), 0.825 (95% CI, 0.758–0.894), and 0.804 (95% CI, 0.730–0.882), respectively (Additional file 1: Fig. S1).

### Heatmaps for interpreting CLA-HDM decision-making

**Fig. 4:**
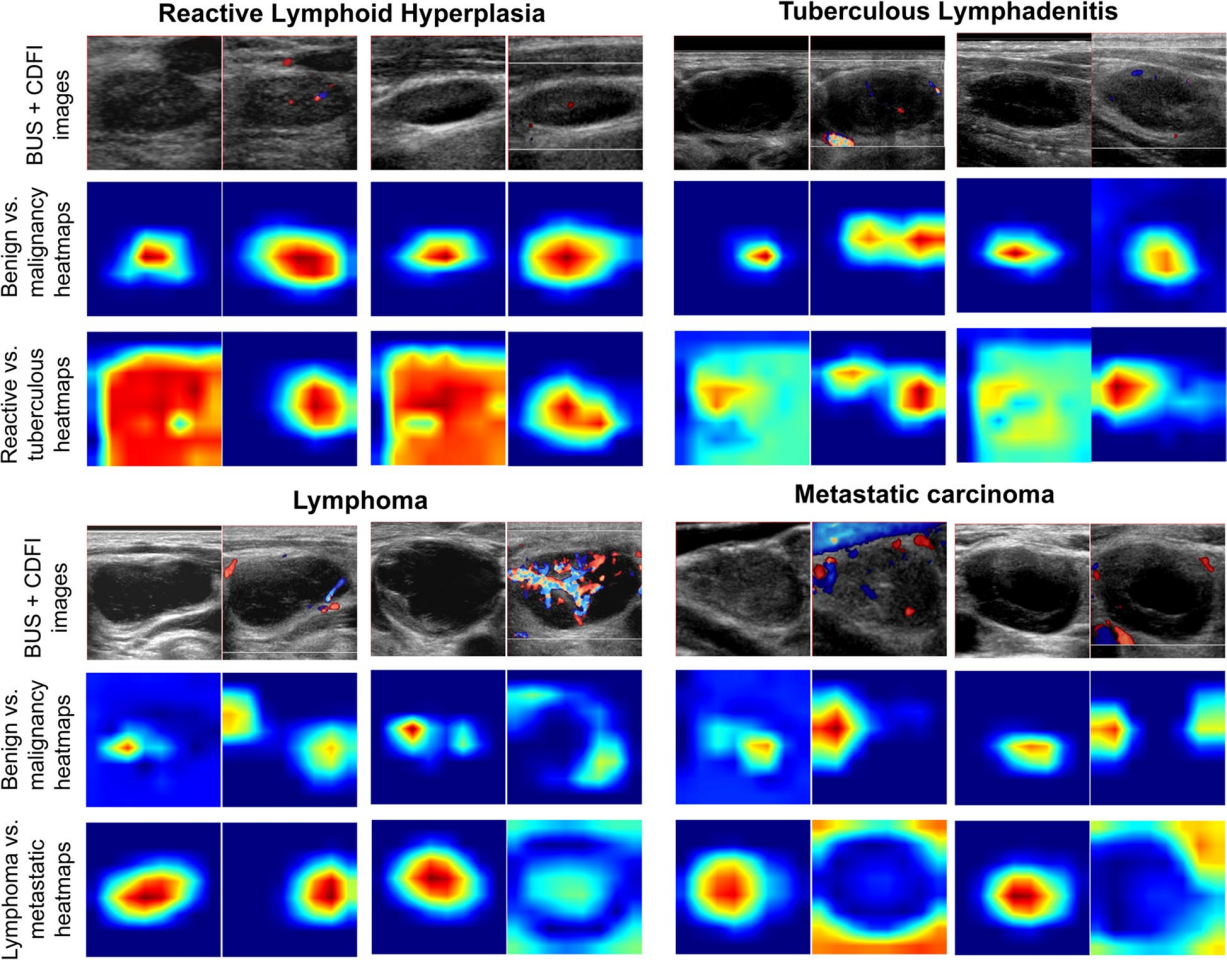
Examples of heatmaps generated by CLA-HDM for each etiology of unexplained CLA. When ultrasound BUS and CDFI images of a case (first row) are input into CLA-HDM, it will firstly give first-level diagnostic heatmaps to distinguish benign from malignant CLA (second row) and then second-level diagnostic heatmaps to identify the specific etiologies of benign or malignant CLA (third row). Generally, the heatmaps reveals a corresponding regularity for each pathology category. CLA, cervical lymphadenopathy; BUS, B-mode ultrasound; CDFI, color Doppler flow imaging

After using heatmaps to visualize the decision-making of CLA-HDM, we found clearly different patterns for four etiologies in BUS and CDFI images (Fig. 4). To determine benign or malignancy, model tended to focus on the intranodal region in BUS, which is the same region as radiologists making diagnosis. Heatmaps showed that CLA-HDM concentrated on intranodal vessels, not surrounding vessels for benign CLA in CDFI. However, for malignant CLA, it focused more closely on peripheral or mixed vascularity. Furthermore, the focus on CDFI tended towards the most abundant intranodal vessels for reactive hyperplasia, but towards the peripheral vessels for tuberculosis. Differently, when CLA-HDM successfully identified lymphoma, it focused on the area of intense hilar vascularity in CDFI, but it paid attention to the surrounding peripheral area for the true positive diagnosis of metastatic carcinoma, forming a lollipop shape in CDFI. Those information was notified to radiologists for diagnosis assistance in this study.

### First stage of the radiologist study

**Fig. 5:**
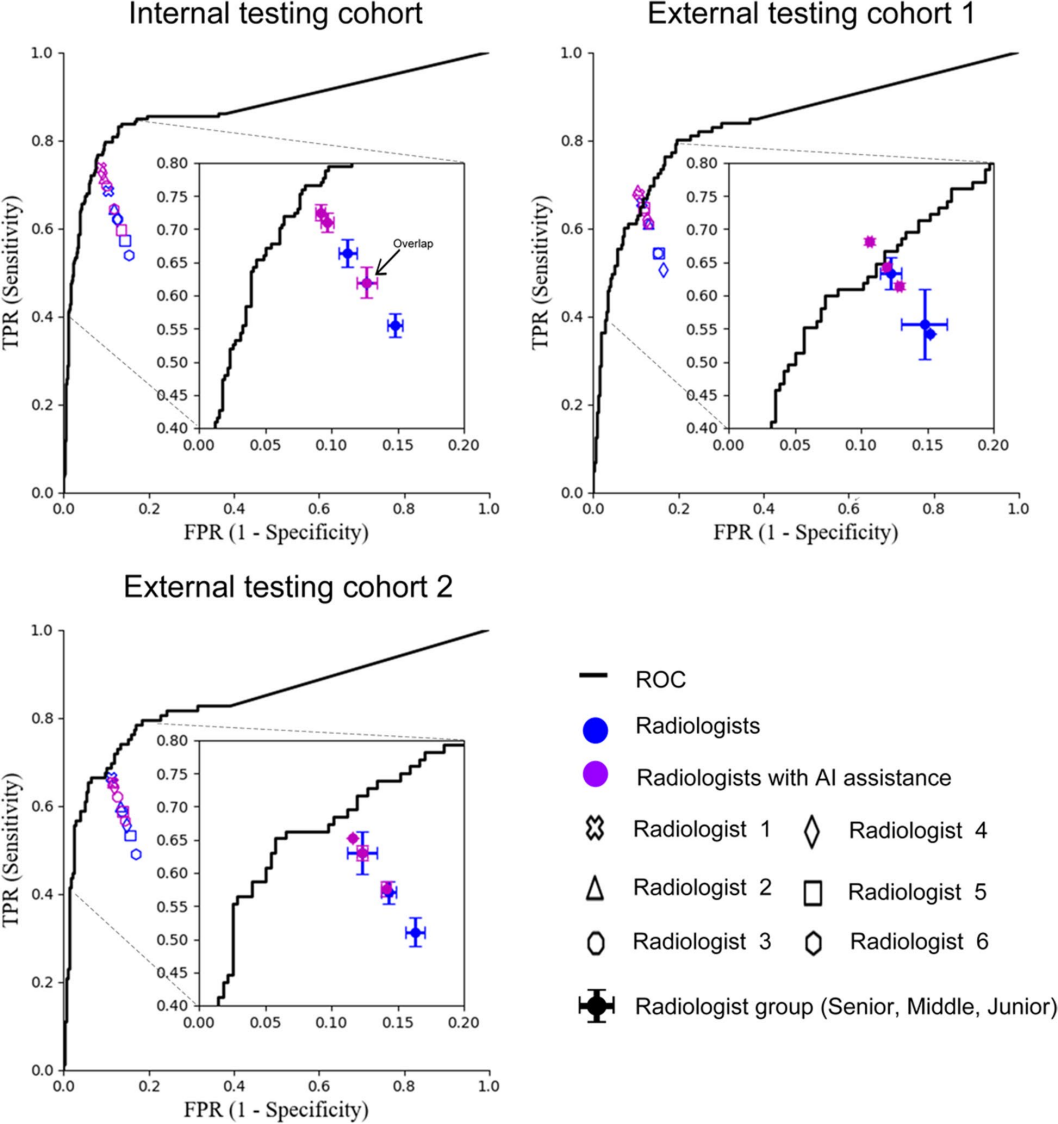
Comparison between CLA-HDM and radiologists and between radiologists without and with AI assistance to identify four common etiologies for unexplained CLA. Radiologists 1 and 2 represent senior-level experience, radiologists 3 and 4 represent middle-level experience, and radiologists 5 and 6 represent junior-level experience. ROC, receiver operating characteristic curve; AI, artificial intelligence; CLA, cervical lymphadenopathy

In the first stage, six radiologists without AI assistance and CLA-HDM were recruited for the radiologist-machine comparison. Compared with each individual radiologist, CLA-HDM achieved systematically better accuracy, sensitivity, and specificity than all radiologists in the three testing cohorts, except for radiologist 1 (a senior radiologist) in the external testing cohort 1, who had equivalent performance to the CLA-HDM (*P* >.05, Fig. 5 and Table 2). Moreover, CLA-HDM showed significantly better accuracy, sensitivity, and specificity than some of these radiologists in different testing cohorts (*P* < 0.05, Table 2). Compared with three different levels of radiologist groups, CLA-HDM also achieved systematically better accuracy, sensitivity, and specificity than all groups and was significant in at least one testing cohort (*P* < 0.05, Fig. 5 and Table 3).

**Table 2:** Comparison of diagnostic performance between CLA-HDM and six radiologists, and between radiologists with and without AI assistance

Radiologists		Internal testing cohort (*n* = 171)		External testing cohort 1 (*n* = 105)		External testing cohort 2 (*n* = 92)	
		Without AI (%)	With AI (%)	Without AI (%)	With AI (%)	Without AI (%)	With AI (%)
1	Accuracy	84.2 (81.3, 87.4)	86.8 (83.9, 89.8)↑#	82.9 (79.1, 86.7)	83.8 (80.0, 87.6)↑	83.2 (78.8, 87.0)	82.6 (78.3, 86.4)
	Sensitivity	68.4 (62.6, 74.9)	73.7 (67.8, 79.5)↑	65.7 (58.1, 73.3)	67.6 (60.0, 75.2)↑	66.3 (57.6, 73.9)	65.2 (56.5, 72.8)
	Specificity	89.5 (87.5, 91.6)	91.2 (89.3, 93.2)↑	88.6 (86.0, 91.1)	89.2 (86.7, 91.8)↑	88.8 (85.9, 91.3)	88.4 (85.5, 90.9)
2	Accuracy	82.2 (79.5, 85.1) **	85.7 (83.0, 88.3)↑##	80.5 (76.7, 84.8)	84.3 (80.5, 88.1)↑#	79.9 (76.1, 84.2)	82.6 (78.8, 87.0)↑
	Sensitivity	64.3 (59.1, 70.2) *	71.4 (66.1, 76.6)↑##	60.9 (53.3, 69.5)	68.6 (61.0, 76.2)↑	59.8 (52.2, 68.5)	65.2 (57.6, 73.9)↑
	Specificity	88.1 (86.4, 90.1)	90.5 (88.7, 92.2)↑	86.9 (84.4, 89.8)	89.5 (87.0, 92.1)↑	86.6 (84.1, 89.5)	88.4 (85.9, 91.3)↑
3	Accuracy	81.0 (78.1, 83.9) **	84.8 (82.2, 87.7)↑##	80.5 (76.2, 84.8)	80.5 (76.7, 84.3)↑	79.4 (75.0, 83.7)	81.0 (76.6, 85.3)↑
	Sensitivity	62.0 (56.1, 67.8) *	69.6 (64.3, 75.4)↑#	60.9 (52.4, 69.5)	61.0 (53.3, 68.6)↑	58.7 (50.0, 67.4)	62.0 (53.3, 70.7)↑
	Specificity	87.3 (85.4, 89.3) *	89.9 (88.1, 91.8)↑	87.0 (84.1, 89.8)	87.0 (84.4, 89.5)↑	86.2 (83.3, 89.1)	87.3 (84.4, 90.2)↑
4	Accuracy	81.0 (78.1, 84.2) **	86.3 (83.6, 89.2)↑###	75.2 (71.4, 79.5) **	81.0 (77.1, 84.8)↑##	77.7 (73.9, 82.2)	82.1 (78.3, 86.4)↑#
	Sensitivity	62.0 (56.1, 68.4) *	72.5 (67.3, 78.4)↑###	50.5 (42.9, 59.1)*	61.9 (54.3, 69.5)↑#	55.4 (47.8, 64.1) *	64.1 (56.5, 72.8)↑
	Specificity	87.3 (85.4, 89.5) *	90.8 (89.1, 92.8)↑	83.5 (81.0, 86.4)	87.3 (84.8, 89.8)↑	85.1 (82.6, 88.0)	88.0 (85.5, 90.9)↑
5	Accuracy	78.7 (75.4, 81.9) ***	79.8 (76.9, 83.0)↑	77.1 (72.9, 81.0) *	82.4 (78.6, 86.7)↑#	76.6 (72.3, 81.0)	79.4 (75.5, 84.2)↑
	Sensitivity	57.3 (50.9, 63.7) **	59.7 (53.8, 66.1)↑	54.3 (45.7, 61.9)	64.8 (57.1, 73.3)↑#	53.3 (44.6, 62.0) *	58.7 (51.1, 68.5)↑
	Specificity	85.7 (83.6, 87.9) **	86.6 (84.6, 88.7)↑	84.8 (81.9, 87.3)	88.3 (85.7, 91.1)↑	84.4 (81.5, 87.3) *	86.2 (83.7, 89.5)↑
6	Accuracy	76.9 (73.7, 80.1) ***	82.2 (78.9, 85.1)↑##	77.1 (73.3, 81.0) *	81.9 (78.1, 86.2)↑#	74.5 (70.1, 78.8) *	78.3 (73.9, 82.6)↑
	Sensitivity	53.8 (47.4, 60.2) ***	64.3 (57.9, 70.2)↑##	54.3 (46.7, 61.9)	63.8 (56.2, 72.4)↑	48.9 (40.2, 57.6) **	56.5 (47.8, 65.2)↑
	Specificity	84.6 (82.5, 86.7) **	88.1 (85.9, 90.1)↑	84.8 (82.2, 87.3)	87.9 (85.4, 90.8)↑	83.0 (80.1, 85.9) **	85.5 (82.6, 88.4)↑

The data in brackets represent the 95% confidence intervals. * indicates a statistically significant difference between CLA-HDM and radiologist without AI assistance (*P < 0.05, **P < 0.01, and ***P < 0.001); # indicates a statistically significant difference between radiologist without and with CLA-HDM assistance (^#^*P* < 0.05, ^##^*P* < 0.01, and ^###^*P* < 0.001). The upward arrow (↑) represents indicators that improved owing to AI assistance

**Table 3:** Comparison of diagnostic performance between the groups of radiologists at different levels

Different levels of radiologist group		Internal testing cohort (*n* = 171)			External testing cohort 1 (*n* = 105)			External testing cohort 2 (*n* = 92)		
		Without → with AI (%)	P_1_	P_2_	Without → with AI (%)	*P*_1_	*P*_2_	Without → with AI (%)	*P*_1_	*P*_2_
Senior	Accuracy	83.2 → 86.3 ↑	0.033	/	81.7 → 84.1 ↑	0.132	/	81.5 → 82.6 ↑	0.291	/
	Sensitivity	66.4 → 72.5 ↑	0.032	/	63.3 → 68.1↑	0.132	/	63.0 → 65.2 ↑	0.314	/
	Specificity	88.8 → 90.8 ↑	0.040	/	87.8 → 89.4 ↑	0.116	/	87.7 → 88.4 ↑	0.292	/
Middle	Accuracy	81.0 → 85.5 ↑	0.007	0.534	77.9 → 80.7 ↑	0.088	0.616	78.5 → 81.5 ↑	0.096	0.628
	Sensitivity	62.0 → 71.1 ↑	0.005	0.551	55.7 → 61.4 ↑	0.107	0.614	57.1 → 63.1 ↑	0.087	0.647
	Specificity	87.3 → 90.4 ↑	0.004	0.537	85.2 → 87.1 ↑	0.093	0.589	85.7 → 87.7 ↑	0.102	0.644
Junior	Accuracy	77.8 → 81.0 ↑	0.033	0.420	77.1 → 82.1 ↑	0.013	0.670	75.6 → 78.8 ↑	0.075	0.783
	Sensitivity	58.8 → 62.0 ↑	0.037	0.440	54.3 → 64.3↑	0.009	0.702	51.1 → 57.6 ↑	0.091	0.790
	Specificity	85.2 → 87.3 ↑	0.034	0.430	84.8 → 88.1 ↑	0.016	0.671	83.7 → 85.9 ↑	0.080	0.759

*P*_1_ values indicate a comparison between the AI model and the different levels of radiologist groups without AI assistance. *P*_2_ values indicate a comparison between junior and middle experienced radiologist group with AI assistance and senior experienced radiologist group without AI assistance. The upward arrow (↑) represents indicators that improved owing to AI assistance

### Second stage of the radiologist study

In the second stage, all radiologists in the three testing cohorts achieved higher accuracy, sensitivity, specificity with AI assistance, except for radiologist 1 (a senior radiologist) in the external testing cohort 2, who had a slightly decreased performance, but not significant (*P* >.05, Fig. 5 and Table 2). Specifically, each individual radiologist with AI-assisted had an equivalent or slightly increased specificity, while accuracy and sensitivity were significantly improved in at least one testing cohort (*P* < 0.05, Table 2). In general, we found that in all three testing cohorts, CLA-HDM helped most radiologists to improve their original diagnosis, especially for reactive hyperplasia and metastatic carcinoma. Positive and negative examples of the two-stage AI-assistance study were illustrated in Additional file 1: Fig. S2, S3.

By analyzing AI assistance in terms of different radiologist groups, we found that accuracy, sensitivity, and specificity in three testing cohorts were all improved, especially for the junior and middle experience groups, whose improved diagnostic performance was comparable to that of the senior experience group without AI assistance (*P* > 0.05, Fig. 5 and Table 3). Moreover, a reduction in the false-positive rate (0.7–3.1%) and false-negative rate (2.2–10%) in the three groups was observed (Additional file 1: Fig. S4a). If only benign and malignant differentiation of CLA was considered, the false-negative rate of the radiologist groups with AI assistance decreased by 3.5–13.2%, and the false-positive rate decreased by 7.6–14.8% (Additional file 1: Fig. S4b).

## Discussion

In this multi-center study, we proposed a DL model named CLA-HDM for accurately diagnosing unexplained CLA by integrating BUS and CDFI images. After both internal and external independent validations, it was proven to be effective in assisting radiologists, with a systematic improvement of their diagnostic accuracy in classifying unexplained CLA into reactive hyperplasia, tuberculous lymphadenitis, metastatic carcinoma, and lymphomas. It was especially helpful for radiologists with junior and intermediate experience. With AI assistance, their diagnoses were improved to the similar level of senior radiologists. To the best of our knowledge, this is the first study that uses a DL based radiomics model with medical images for the characterization of unexplained CLA patients. In total, 763 patients from three hospitals participated in this study, which guaranteed its credibility and provided a good basis for initiating larger scale perspective investigations in future.

CLA-HDM did not only provide a clinical judgement of unexplained CLA, but also visualized its decision-making by key feature-based heatmaps. By interpreting these heatmaps with senior physicians, we found that they often showed distinct and recognizable patterns for different etiologies. For the BUS images, there were two locations valuable for CLA-HDM to diagnose unexplained CLA, namely the lesion margins and the internal echoes of the lymph nodes; for the CDFI images, the model focused on the locations of the vasculature. This was consistent with the clinical experience and relevant studies [12, 43–45]. Specifically, malignant CLAs were typically associated with distinct features, such as well-defined sharp margins, extensive intranodal structural variations (for example, intranodal necrosis is common in metastases and reticulation is common in lymphomas), and abundant peripheral vascularity [43, 44]; the highlighted regions in the heatmaps were helpful to identify these representative characteristics of malignant CLAs. However, in most benign CLAs, the margins were ill-defined and blurry, the intranodal structure changed slightly, and vessels were rarely or only detected intranodal vessels (for example, avascular or hilar vascular flow is common in reactive CLAs and displacement vessel is common in tuberculosis) [45, 46]. And as a result, the entire lymph node and its peripheral areas on BUS images and intranodal vessels on CDFI images of benign CLAs is of importance in AI interpretation. These patterns were also consistent with biological or pathological characteristics of each etiology, which give a good direction for further investigation, but such speculations still need direct evidence to confirm. However, heatmaps undoubtedly played a good role in guiding radiologists, especially when they were facing some challenging cases with non-negligible uncertainty. This effective assistance was positively confirmed by all radiologists involved.

Compared with other studies of classifying malignant draining lymph node metastasis [47, 48], our study is facing a more complex clinical scenario, but the proposed model still achieved a good performance in both dichotomous and quadruple classification of unexplained CLA. More importantly, it was proved to be a good assisting tool for radiologists to improve their overall diagnostic accuracy. It revealed a great potential of helping radiologists to avoid subjective bias related to professional experience, which may reduce unnecessary investigations, inappropriate or delayed treatments. This especially holds a big significance for CLA patients in underdeveloped countries and regions.

There are several limitations in this study. First, the dataset we used for model development had a category imbalance across etiologies of unexplained CLA. This is mainly due to differences in the prevalence and clinical management of each etiology. When clinicians consider patients with unexplained CLA to be benign cases, they generally use follow-up rather than invasive procedures, resulting in a relatively small proportion of benign CLA cases of 34.4% included in the study. Also, the low prevalence of lymphoma compared to metastatic carcinoma resulted in a significant category imbalance within the group of malignant CLA. These factors affect the diagnostic performance of the model to some extent, and using more and broader data to address this issue will be an important direction for future work. Second, the retrospective nature of this study caused inevitable deviations. Our future research will incorporate the AI system into routine clinical workflows for perspective validations. Finally, the patients in this study were from medically underdeveloped regions of China. Therefore, the proposed model needs to undergo a multi-region survey for a more comprehensive investigation.

## Conclusions

The proposed CLA-HDM based on dual-modality ultrasound images showed systematically better accuracy, sensitivity, and specificity in the diagnosis of four common etiologies of unexplained CLA than skilled radiologists. It helped to narrow the gap between radiologists with different levels of experience in classification, which is potentially of great significance for CLA patients in underdeveloped countries and regions.

## Supplementary Information

**Additional file 1: Method S1.** Data collection and preprocessing. **Method S2.** Structure of our model. **Method S3.** Strategy of training our model. **Method S4.** Measuring the performance of our model. **Method S5.** Visualization of our model. **Table S1.** Detailed make and model of ultrasound diagnostic instrument used in the study. **Table S2.** Summary of histological types of cervical lymphadenopathy. **Table S3.** Baseline characteristics in the training and testing cohorts. **Table S4.** The structure and hyper-parameters of CLA-HDM sub-models. **Figure S1**. Diagnostic performance of CLA-HDM and six individual radiologists for four specific etiologies of CLA in testing cohorts. **Figure S2**. Typical cases of CLA-HDM guiding radiologists to make correct decisions. **Figure S3**. Typical cases of CLA-HDM that misled radiologists to make incorrect decisions. **Figure S4**. Diagnostic performance of different levels of radiologist groups before and after AI-assistance.

## Data Availability

Clinical and ultrasound images are not publicly available to protect patient privacy. Original images may be made available upon reasonable request to the corresponding authors (F.N. and K.W.). The code is available at https://github.com/RichardSunnyMeng/CLA-HDM.

## Abbreviations

AI: Artificial intelligence
AUC: Areas under the curve
BUS: B-mode ultrasound
CDFI: Color Doppler flow imaging
CLA: Cervical lymphadenopathy
CLN: Cervical lymph node
DL: Deep-learning
ROC: Receiver operating characteristic curve
ROI: Regions of interest
