# Supplementary material for: Deep learning radiomics of dual-modality ultrasound images for hierarchical diagnosis of unexplained cervical lymphadenopathy
Source: BMC Med. 2022 Aug 26;20:269. doi: 10.1186/s12916-022-02469-z (PMC9410737; doi:10.1186/s12916-022-02469-z)
Supplement: Supplementary file 1 — Additional file 1: Method S1. Data collection and preprocessing. Method S2. Structure of our model. Method S3. Strategy of training our model. Method S4. Measuring the performance of our model. Method S5. Visualization of our model. Table S1. Detailed make and model of ultrasound diagnostic instrument used in the study. Table S2. Summary of histological types of cervical lymphadenopathy. Table S3. Baseline characteristics in the training and testing cohorts. Table S4. The structure and hyper-parameters of CLA-HDM sub-models. Figure S1. Diagnostic performance of CLA-HDM and six individual radiologists for four specific etiologies of CLA in testing cohorts. Figure S2. Typical cases of CLA-HDM guiding radiologists to make correct decisions. Figure S3. Typical cases of CLA-HDM that misled radiologists to make incorrect decisions. Figure S4. Diagnostic performance of different levels of radiologist groups before and after AI-assistance. [file 12916_2022_2469_MOESM1_ESM.docx]

# Additional file 1

# Supplementary Material

## Methods

Method S1. Data collection and preprocessing.

Method S2. Structure of our model.

Method S3. Strategy of training our model.

Method S4. Measuring the performance of our model.

Method S5. Visualization of our model.

## Tables

Table S1. Detailed make and model of ultrasound diagnostic instrument used in the study.

Table S2. Summary of histological types of cervical lymphadenopathy.

Table S3. Baseline characteristics in the training and testing cohorts.

Table S4. The structure and hyper-parameters of CLA-HDM sub-models.

## Figures

Fig. S1. Diagnostic performance of CLA-HDM and six individual radiologists for four specific etiologies of CLA in testing cohorts.

Fig. S2. Typical cases of CLA-HDM guiding radiologists to make correct decisions.

Fig. S3. Typical cases of CLA-HDM that misled radiologists to make incorrect decisions.

Fig.S4. Diagnostic performance of different levels of radiologist groups before and after AI-assistance.

**Methods 1. Data collection and preprocessing**

**Data collection**

In this study, two radiologists in each center collaborated to perform the collection of ultrasound images and were prohibited from participating in the subsequent study. Specifically, one radiologist initially collected all ultrasound images of patients eligible for the study based on clinical features and pathological findings. All these ultrasound data were then handed over to another radiologist, who further screens ultrasound images centering only on image quality, without knowing pathological and clinical information. In this way, poor-quality ultrasound images such as those with motion artifacts or incomplete lymph node sections can be excluded, and the influence of the doctor's subjective perception and objective ability on the data set during image collection can be effectively avoided.

**Data preprocessing**

In deep-learning, prior knowledge refers to integrating our cognition of a specific task into the design or training of the model so that the model can be trained spontaneously in the desired direction. In this study, we used B-mode ultrasound (BUS) and color Doppler flow imaging (CDFI) to diagnose cervical lymphadenopathy (CLA) in patients. In a BUS or CDFI image, there are often many interfering factors around the lymph nodes, such as other nearby lymph nodes and their blood flow. Therefore, two experienced radiologists who were blinded to pathology findings and clinical information were involved in the generation of the region of interests (ROIs): first, a radiologist with 10 years of ultrasound experience read the pathology-confirmed CLA ultrasound images and completed annotations of the ROIs by individually depicting the relevant cervical lymph node boundaries in each BUS and CDFI image. Subsequently, the above annotated results were examined by another radiologist with 20 years of ultrasound experience. If there was a dispute, they reached consensus through discussion to ensure the correctness of the segmentations. In this way, prior knowledge can be introduced to eliminate the influence of interfering factors from the surrounding area. Finally, the input of the model was the smallest circumscribed square with RoI as the mask.

Data from hospital 1 were used as the primary cohort to reduce overfitting or bias in the analysis. Lesions prior to 2021 were selected in the primary cohort as the training cohort for model development, while lesions from 2021 were used as the internal testing cohort to simulate prospective experimental conditions. Data from hospital 2 and hospital 3 were used as independent external testing cohorts. Before feeding forward into the model, all inputs were standardized to a mean of 0 and a variance of 1, which is a common processing method for image data in deep-learning. The formula for standardizing image data is as follows:

$$I_{i}^{'}\left( x, y \right)= \frac{I_{i}\left( x, y \right)-\mu}{\sigma}$$

where $I_{i}\left( x, y \right)$ denotes the pixel value of the original image $I_{i}$ at (x, y) (normalized to [0, 1]), $I_{i}^{'}\left( x, y \right)$ denotes the pixel value of the standardized image $I_{i}^{'}$ at (x, y). $\mu$ denotes the mean value, and $\sigma$ denotes the variance of the entire image following the formula below:

$$\mu= \frac{1}{K}\sum_{k=1}^{K} \frac{1}{M \times N}\sum_{x=0}^{M-1} \sum_{y=0}^{N-1} I_{k}(x, y)$$

$$\sigma^{2} = \frac{1}{K}\sum_{k=1}^{K} \frac{1}{M \times N}\sum_{x=0}^{M-1} \sum_{y=0}^{N-1} \left( I_{k}\left( x, y \right)-\mu\right)^{2}$$

where K denotes the number of training images. The above standardization method was employed for each channel of inputs.

Data augmentation is another common method to increase the size of the dataset so that the model can learn more features with invariant characteristics to prevent the occurrence of overfitting, particularly in few-shot learning. Commonly used data augmentation methods include random vertical and horizontal flipping, random rotation, and random cropping. To maintain the integrity of the area of the node and its surroundings, we used only the first two in the training stage. In this study, we set the vertical and horizontal flipping probabilities to 0.5, and the random rotation range was [-60°, 60°]. In other words, the input of the model will flip vertically and horizontally with a probability of 0.5, and rotate an angle randomly within the range of -60°to 60°. Note that the US and CDFI inputs of the same case were augmented with the same hyper-parameters. Finally, we resized all inputs to (256, 256) so that we could train our model in parallel to obtain a better performance.

*2D convolution*

The convolution has been proven to be one of the most effective tools for extracting features of high-dimensional data, particularly local features. Through layer-by-layer stacking of the convolutional layer, high-level semantic features can be effectively extracted. The convolution formula is as follows:

$$f\left( x,y \right)= \sum_{m=-k_{w}}^{k_{w}} \sum_{n=-k_{h}}^{k_{h}} I(x+m, y+n)k(m,n)$$

where $I$ denotes the input image and f is the output image (also called the feature map). $k$ is the learnable convolution kernel, and $k_{w}$ and $k_{h}$ are the width and height of $k$.

In this study, we utilized the residual network (ResNet) [32], a typical deep convolution neural network (DCNN) to extract features. This is a network structure with multiple cascaded residual convolutional blocks. The residual connected structure can effectively address the difficulty of training a deep network and improve the performance of the model. More specifically, ResNet-50, a type of ResNet with 50 layers, was used in our model.

*Rectified linear unit*

To imitate the work of brain neurons and enhance the model's ability to fit nonlinear functions, an activation function usually follows each layer in deep models. The rectified linear unit (ReLU) [33] is a commonly used activation function. The mathematical definition of ReLU is as follows:

$$\mathrm{ReLU}\left( x \right)=max(0, x)$$

The biggest characteristic of ReLU is that it not only introduces nonlinearity but also maintains a gradient of 1 in the interval of x > 0, which effectively avoids the phenomenon of gradient explosion and gradient disappearance in other activation functions such as sigmoid.

In our study, we applied ReLU as the activation function of each layer, except for the last layer. In the last layer, we applied the softmax function as an activation function to output probabilities. The mathematical definition of softmax is as follows:

$$\mathrm{Softmax}\left( x \right)= \frac{e^{x}}{\sum_{m} e^{m}}$$

**Batch normalization**

Batch normalization (BN) [34] is used to solve the difficulty of training caused by the continuous change in the distribution parameters of the data during the training process in DL. As mentioned above, we standardized the inputs to mean 0 and variance 1. However, during training, the parameters of the model change. For a certain layer, the distribution of inputs captured by the model in the last training epoch may not be consistent with that in the next training epoch. Changing the data distribution increases the difficulty of training. Owing to the usage of the GPU, we trained the model in parallel with multiple inputs, called mini-batch, to accelerate the training procedure. We used mini-batch to continuously update the distribution parameters of inputs and standardize inputs to mean 0 and variance 1. Specifically, in the training phase, we calculated the mean and variance of the data in the minibatch according to the following formula and updated the total mean and total variance.0

$$\mu_{B}= \frac{1}{m}\sum_{k=1}^{m} x_{k}$$

$$\sigma^{2}= \frac{1}{m}\sum_{k=1}^{m} \left( x_{k}-\mu_{B} \right)^{2}$$

Then, we standardize the inputs:

$$\hat{x}= \frac{x-\mu_{B}}{\sqrt{\sigma^{2}+ \epsilon}}$$

where $\epsilon$ is a small number used to stabilize the output. For the model to learn a distribution that is most conducive to subsequent tasks, let $\gamma$ and $\beta$ be learnable parameters, and the final output is:

$$y=\gamma\hat{x}+\beta$$

In this study, we followed the ResNet-50 structure and placed BN after each convolution layer.

**Global average pooling**

Global average pooling (GAP) is one of the methods used to aggregate global features in deep-learning models. Usually, aggregating global features can be achieved by global average pooling and global maximum pooling. Compared to another, global average pooling can synthesize individual features in the original feature map, so we apply it to the channel attention module and the modality fusion attention module in our model DL-DMLDM. As the name suggests, the global average pooling averages over global features:

$$\mathrm{GAP}(f)= \frac{1}{M}\sum_{x} f(x)$$

where $f(x)$ denotes a feature map while M is the number of features in $f(x)$.

**Fully connected**

In classification or regression tasks, the fully connected layer (FC) can usually act as an output layer at the end of the model [35]. In this study, we applied the FC layer to the classification head, auxiliary supervision head, channel attention, and modality fusion attention to obtain probabilities or normalized weights.

**Attention mechanism**

The proposal of the attention mechanism [36, 37] effectively compensates for the shortcoming that the convolution operation can only capture the dependence of local features, and provides a way for extracting global features. To decrease the number of parameters, we adopted a simpler attention structure, that is, a squeeze-and-excitation-like structure [38]. This simply achieves the output of weights through global average pooling, fully connected layer plus ReLU, and softmax activation layer.

In the channel attention block, we used the attention mechanism to generate weights representing the attention level to the R, G, and B channels of the CDFI image, thereby highlighting the blood flow information that is beneficial to the final task. In the modality fusion attention block, the attention mechanism was used to learn the weights of attention to different channels of features in the CDFI branch through the BUS branch so that the US branch could guide the extraction and application of CDFI features.

**Methods 2. Structure of our model**

In this study, we proposed a hierarchical diagnosis model for unexplained CLA, Cervical Lymphadenopathy Hierarchical Diagnosis Model (CLA-HDM), which consisted of three task-specific sub-models (sub-model 1 for the diagnosis of benign and malignant, sub-model 2 for the diagnosis of tuberculous and reactive, and sub-model 3 for the diagnosis of metastatic and lymphoma unexplained CLA). Each sub-model had a dual-branch structure to fuse dual-modality image data and used attention mechanism to enhance the attention to different color blood flow information and to different channel features of CDFI. The design philosophy of modality fusion attention and channel attention block has been discussed in the main text. The hyperparameters related to the model structure are presented in Supplementary eTable 4.

CLA-HDM determined the specific pathological type based on the diagnosis conclusion of benign and malignant. Specifically, let the probability of malignant by sub-model 1 be $p_{1}$, the probability of tuberculous and reactive by sub-model 2 be $p_{2}\left( \mathrm{Tuberculous} \right)$and $p_{2}(Reactive)$, the probability of lymphoma and metastatic by sub-model 3 be $p_{3}(Lymphoma)$ and $p_{3}(Metastatic)$, respectively. Then the probability for each pathological type by CLA-HDM on each pathological type is:

$$p(Reactive)=\left\{ \begin{aligned} p_{2}(Reactive) if p_{1}\leq thr \\ 0 if p_{1}> thr \end{aligned} \right.$$

$$p(Tuberculous)=\left\{ \begin{aligned} p_{2}(Tuberculous) if p_{1}\leq thr \\ 0 if p_{1}> thr \end{aligned} \right.$$

$$p(Lymphoma)=\left\{ \begin{aligned} 0 if p_{1}\leq thr \\ p_{3}(Lymphoma) if p_{1}> thr \end{aligned} \right.$$

$$p(Metastatic)=\left\{ \begin{aligned} 0 if p_{1}\leq thr \\ p_{3}(Metastatic) if p_{1}> thr \end{aligned} \right.$$

where $thr$ was Youden threshold.

In addition, we trained a model with the same structure to directly diagnose four common etiologies of unexplained CLA. The results show a similar performance to the respective AUC, but the few-shot category (such as lymphoma) is completely submerged. Therefore, we did not adopt this structure.

**Methods 3. Strategy of training our model**

As highlighted in the main text, a multi-supervision method was adopted to train our sub-model. The loss function is composed of three terms:

$$Loss\left( y, \hat{y} \right)=\lambda{Loss}_{1}\left( y, \hat{y} \right)+\beta Loss_{2}\left( y, \hat{y} \right)+\beta Loss_{3}\left( y, \hat{y} \right)$$

where $y$ denotes a true label, $\hat{y}$ denotes the corresponding prediction score, and $\lambda, \beta$ are hyper-parameters and are set to 1 and 0.5, respectively. $\mathrm{Loss}_{1}$, $\mathrm{Loss}_{2}$, and $\mathrm{Loss}_{3}$ are all cross-entropy functions used to supervise the parameter learning of the entire network, the BUS branch, and the CDFI branch. Reweighting is also applied to these three loss functions to compensate for the impact of category imbalance on training:

$$\mathrm{Loss}_{i}\left( y, \hat{y} \right)= -(\omega_{2}y\log\left( \hat{y} \right)+\omega_{1}\left( 1-y \right)\log\left( 1-\hat{y} \right) )$$

where $\omega_{1}$ and $\omega_{2}$ are the proportions of the positive and negative samples, respectively. Let N be the total number of samples, and the total loss function used to train the model is:

$$\mathcal{L}= \frac{1}{N}\sum_{n=1}^{N} Loss(y_{n}, \hat{y}_{n})$$

We adopted a three-stage transfer learning strategy. In the first stage, we pretrained ResNet-50 using 1.28 million natural images on the ImageNet dataset. Then, the parameters of ResNet-50, except for the fully connected layer, were used to initialize our three sub-models. Finally, these three sub-models were assembled to form CLA-HDM and fine-tuned on our training dataset. As for other blocks such as MFA, channel attention, and three heads, the parameters were initialized using the Xavier initialization method[39] with a gain of 1. The Adam optimizer[40] was used to optimize our model parameters, and the initial learning rate was set to 0.0001 and decayed by $\gamma$(=0.1) once the number of epochs reached one of the milestones([30, 70]). We trained the sub-models for 100 epochs and fine-tuned CLA-HDM for 20 epochs on NVIDIA GeForce GTX 1080Ti under Python 3.8 and Pytorch 1.9.0[41] deep-learning framework. The batch size was set to 32, and early stopping was also adopted. The code will be available at <https://github.com/RichardSunnyMeng/CLA-HDM>.

**Methods 4. Measuring the performance of our model**

In this study, we used the sensitivity, specificity, receiver operating curve (ROC), area under curve (AUC), Youden index, and accuracy to evaluate the performance of CLA-HDM and three sub-models. Let TP, TN, FP, and FN be the numbers of true positive, true negative, false positive, and false negative samples, respectively.

1. Sensitivity, specificity, receiver operating curve (ROC), and area under curve (AUC)

Sensitivity, also known as the true positive rate (TPR), reflects the ability to determine patients. Its mathematical formula is as follows:

$$Sensitivity= \frac{TP}{TP+FN}$$

Specificity, also known as the true negative rate (TNR), reflects the ability to determine non-patients. Its mathematical formula is as follows:

$$Specificity= \frac{TN}{TN+FP}$$

Sensitivity and Specificity are determined under a certain threshold.

The receiver operating curve (ROC) reflects the trade-off between the sensitivity and specificity of the model for patient diagnosis. When drawing the ROC curve, the model prediction value of each patient was used as the threshold, and the sensitivity and specificity of the model results under these thresholds were calculated, and the ROC curve was drawn with 1-Specificity as the horizontal axis and sensitivity as the vertical axis. The area under the curve (AUC) was the area under ROC. The closer the AUC is to 1, the better is the model performance.

1. Youden Index and Accuracy

The mathematical formula of Youden Index is

$$Youden Index=Specificity+Sensitivity-1$$

The larger the index, the better the model performance. At the same time, the Youden index provides an optimal threshold for diagnosis. We calculated the maximum Youden Index of CLA-HDM and three sub-models in the training set as the optimal threshold, and used it to calculate the accuracy, sensitivity, and specificity. The calculation formula for the accuracy is

$$Accuracy= \frac{TP+TN}{TP+FN+TN+FP}$$

**Methods 5. visualization of our model**

In this paper, in order to enhance the interpretability of CLA-HDM, and at the same time provide further help to clinicians to make a correct diagnosis, we visualized the region on both BUS and CDFI images by focusing on the model and the features extracted by the model.

To facilitate observation and understanding, we only show 256 low-level semantic features of the layer before Conv2 on both the BUS and CDFI branches. All features were transformed into pseudo-colored grid maps using the OpenCV toolkit [30]. On the contrary, we set the last convolution layer as the target layer and visualized its output feature map as heatmaps by Layer-CAM [31], where a warmer color (e.g., red and yellow) indicated a stronger correlation with the diagnosis prediction of our model and cooler color (e.g., blue and greed) indicated a weaker correlation region.

**Table S1.** Detailed make and model of ultrasound diagnostic instrument used in the study.

| Research center | Ultrasound Diagnostic Instrument Information | | |
| --- | --- | --- | --- |
|  | Manufacturer | Model | Site |
| Hospital 1  (n= 566) | Philips (n = 411) | IU-22 | Philips Medical Systems, Bothell, USA |
|  | General Electric (n = 63) | Vivid E9 | General Healthcare, Milwaukee, WI, USA |
|  | Siemens (n = 36) | ACUSON SEQUOIA 512 | Siemens Medical Solutions, CA, USA |
|  |  | ACUSON S2000 | Siemens AG, Erlangen, Germany |
|  | Fenoxaparin (n = 28) | Vinno M80 | Vinno, Suzhou, China |
|  |  | Vinno M86 | Vinno, Suzhou, China |
|  | Others (n = 28) | HI VISION Ascendus | Hitachi Ltd, Tokyo, Japan |
|  |  | Esaote MyLab60 | Esaote Group, Italy |
|  |  | TOSHIBA Aplio 300 | Toshiba Medical Systems, Tokyo, Japan |
|  |  | Mindray Resona 7 | Mindray, Shenzhen, China |
|  |  | Aixplorer | SuperSonic Imaging, Aix-en-Provence, France |
| Hospital 2  (n = 105) | Mindray (n = 59) | Resona 7 | Mindray, Shenzhen, China |
|  | Siemens (n = 22) | ACUSON X300 | Siemens Medical Solutions, Mountain View, CA, USA |
|  | Philips (n = 14) | IU Elite | (Philips Healthcare, Andover, MA, USA |
|  | General Electric (n = 10) | Vivid E9 | General Healthcare, Milwaukee, WI, USA |
| Hospital 3  (n = 92) | Philips (n = 47) | IU-22 | Philips Healthcare, Andover, MA, USA |
|  | General Electric (n = 26) | voluson S8 | GE Healthcare, Milwaukee, WI, USA |
|  | Siemens (n = 19) | ACUSON S2000 | Siemens AG, Erlangen, Germany |

**Table S2.** Summary of histological types of cervical lymphadenopathy.

| Cervical lymphadenopathy's histopathologic types | | Hospital 1  (n = 566) | | Hospital 2  (n = 105) | Hospital 3  (n = 92) |
| --- | --- | --- | --- | --- | --- |
| Benign | Total | | 193 | 33 | 37 |
|  | Reactive hyperplasia | | 138 | 21 | 21 |
|  | Tuberculous lymphadenitis | | 55 | 12 | 16 |
| Lymphoma | Total | | 48 | 10 | 8 |
|  | Hodgkin's lymphoma | | 14 | 2 | 2 |
|  | Non-Hodgkin Lymphoma | | 34 | 8 | 6 |
|  | Diffuse large B cell lymphoma | | 21 | 5 | 4 |
|  | Follicular lymphoma | | 2 | 1 | 1 |
|  | Mantle cell lymphoma | | 3 | 1 | / |
|  | Small lymphocytic lymphoma | | 2 | / | / |
|  | Burkitt lymphoma | | 1 | / | 1 |
|  | Anaplastic large cell lymphoma | | 2 | / | / |
|  | Adult T-cell leukemia | | 2 | 1 | / |
|  | Angioimmunoblastic T-cell lymphoma | | 1 | / | / |
| Metastatic | Total | | 325 | 62 | 47 |
|  | Lung | | 97 | 18 | 20 |
|  | Thyroid | | 61 | 10 | 7 |
|  | [Nasopharynx](https://dict.youdao.com/w/nasopharynx/#keyfrom=E2Ctranslation) | | 38 | 6 | 5 |
|  | Stomach | | 33 | 8 | 4 |
|  | Esophagus | | 35 | 5 | 2 |
|  | Breast | | 12 | 3 | 3 |
|  | Larynx | | 6 | / | 3 |
|  | Tongue | | 4 | 1 | / |
|  | Oral cavity | | 3 | 1 | / |
|  | Sarcomatoid carcinoma | | 4 | / | / |
|  | Urothelial carcinoma | | 3 | 1 | / |
|  | Hepatocellular carcinoma | | 2 | / | / |
|  | Melanoma | | 2 | / | 1 |
|  | Sarcoma | | 2 | 1 | / |
|  | Cervix | | 2 | / | 2 |
|  | Prostate | | 2 | / | / |
|  | Pancreas | | 2 | 1 | / |
|  | Colon and rectum | | 2 | 2 | / |
|  | Parotid gland | | 2 | 1 | / |
|  | Ovary | | 1 | 1 | / |
|  | Renal cell carcinoma | | 1 | / | / |
|  | Sinuses | | 1 | / | / |
|  | Tonsil | | 1 | / | / |
|  | Endometrium | | / | 1 | / |
|  | Unknown | | 9 | 2 | / |

**Table S3.** Baseline characteristics in the training and testing cohorts.

| Characteristic | Training cohort  (n = 395) | Internal testing cohort (n = 171) | P_1_ | External testing cohort 1 (n = 105) | P_2_ | External testing cohort 2 (n = 92) | P_3_ |
| --- | --- | --- | --- | --- | --- | --- | --- |
| Age (years)  (mean ± SD) | 51.71 ± 14.17  (16 – 81) | 49.20 ± 13.57  (19 – 81) | 0.525 | 45.41 ± 14.90  (16 – 75) | 0.103 | 44.84±15.06  (18 – 82) | 0.057 |
| Sex |  |  | 0.714 |  | 0.802 |  | 0.898 |
| Male | 209 (52.9%) | 94 (55.0%) |  | 57 (54.3%) |  | 48 (52.2%) |  |
| Female | 186 (47.1%) | 77 (45.0%) |  | 48 (45.7%) |  | 44 (47.8%) |  |
| Location |  |  | 0.625 |  | 0.967 |  | 0.261 |
| Left neck | 189 (47.8%) | 78 (45.6%) |  | 50 (47.6%) |  | 50 (54.3%) |  |
| Right neck | 206 (52.2%) | 93 (54.4%) |  | 55 (52.4%) |  | 42 (45.7%) |  |
| Node LD (cm) | 1.79 ± 0.75 | 1.67 ± 0.75 | 0.057 | 1.84 ± 0.56 | 0.074 | 1.82 ± 0.47 | 0.054 |
| Neck level |  |  | 0.065 |  | 0.114 |  | 0.078 |
| I | 2 (0.5%) | 3 (1.8%) |  | 2 (1.9%) |  | 2 (2.2%) |  |
| Ⅱ | 103 (26.1%) | 29 (17.0%) |  | 29 (27.6%) |  | 25 (27.2%) |  |
| Ⅲ | 80 (20.3%) | 45 (26.3%) |  | 25 (23.8%) |  | 22 (23.9%) |  |
| Ⅳ | 172 (43.5%) | 74 (43.3%) |  | 24 (32.4%) |  | 29 (31.5%) |  |
| Ⅴ | 38 (9.6%) | 20 (11.7%) |  | 15 (14.3%) |  | 14 (15.2%) |  |
| Biopsy |  |  | 0.497 |  | 0.203 |  | 0.631 |
| FNA | 233 (59.0%) | 99 (57.9%) |  | 52 (50.0%) |  | 50 (54.3%) |  |
| CNB | 158 (40.0%) | 72 (42.1%) |  | 51 (49.0%) |  | 41(44.6 %) |  |
| Excision | 4 (1.0%) | 0 (0.0%) |  | 1 (1.0%) |  | 1 (1.1%) |  |

P_1_ indicates the significance between the training and internal testing cohort; P_2_ indicates the significance between the training and external testing cohort 1, and P_3_ indicates the significance between the training and external testing cohort 2. LD, longitudinal diameter. FNA, fine-needle aspiration. CNB, core needle biopsy.

**Table S4.** The structure and hyper-parameters of CLA-HDM sub-models.

| Model | Block | Layer | Parameters |
| --- | --- | --- | --- |
| CLA-HDM | Channel Attention | Global Average Pooling2D | \ |
|  |  | Linear | in_channels=3  out_channels=10 |
|  |  | ReLU | \ |
|  |  | Linear | in_channels=10  out_channels=3 |
|  |  | Softmax | dim=-1 |
|  | Modality Fusion Attention  (MFA) | Linear | in_channels=2048  out_channels=512 |
|  |  | ReLU | \ |
|  |  | Linear | in_channels=512  out_channels=2048 |
|  |  | Softmax | dim=-1 |
|  | Auxiliary Supervision Head  (ASH) | Linear | in_channels=2048  out_channels=256 |
|  |  | ReLU | \ |
|  |  | Linear | in_channels=256  out_channels=2 |
|  |  | Softmax | dim=-1 |
|  | Classification Head | Linear | in_channels=4096  out_channels=512 |
|  |  | ReLU | \ |
|  |  | Linear | in_channels=512  out_channels=2 |
|  |  | Softmax | dim=-1 |
|  | ResNet-50 (without full-connected layers) | Consistent with ResNet-50 | |

**
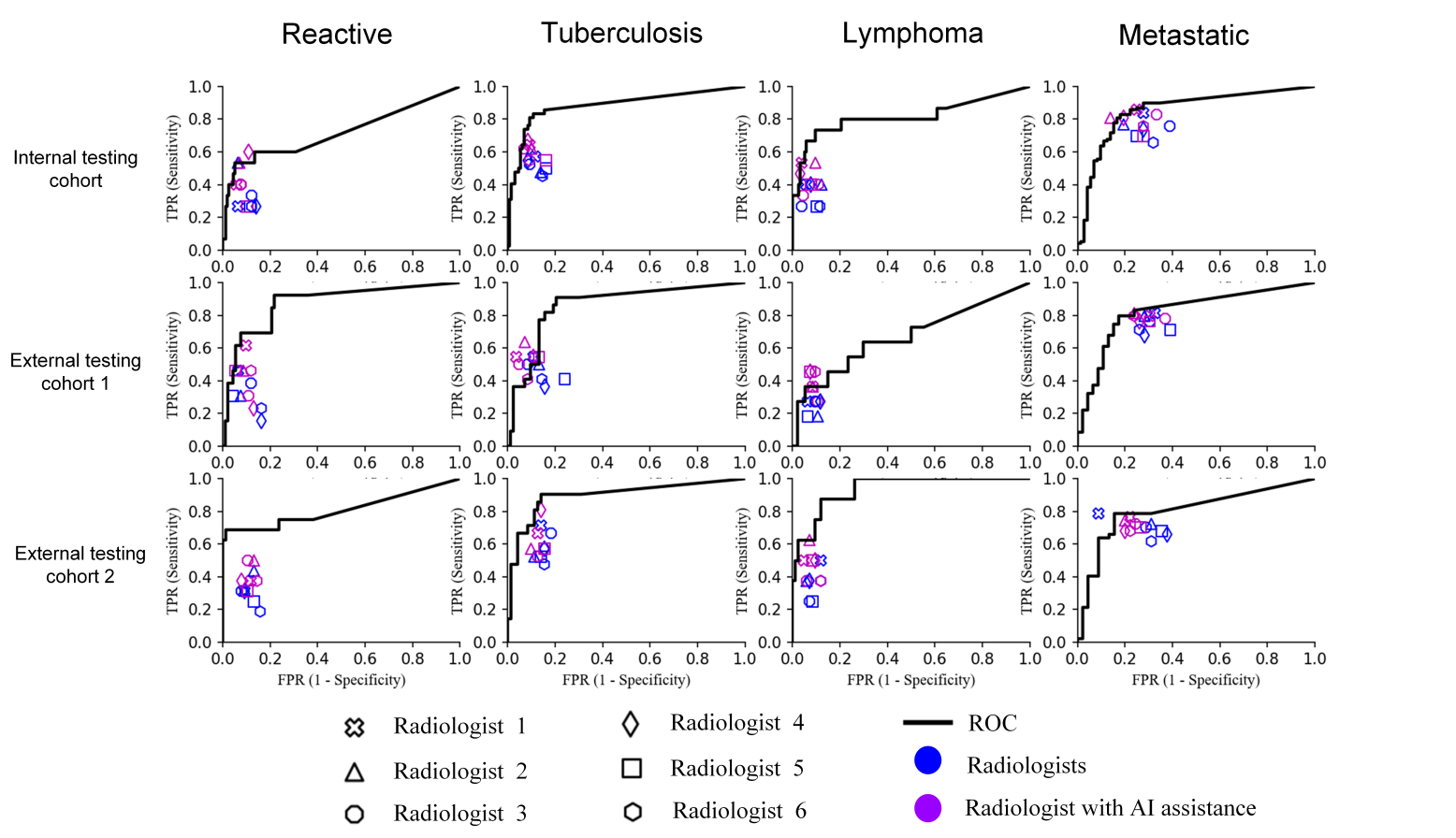
**

**Fig. S1. Diagnostic performance of CLA-HDM and six individual radiologists for four specific pathological types of CLA in testing cohorts.**

Radiologists-1 and -2 represent senior-level experience, radiologists-3 and -4 represent middle-level experience, and radiologists-5 and -6 represent junior-level experience. ROC, receiver operating characteristic curve; AI, artificial intelligence; CLA, cervical lymphadenopathy.


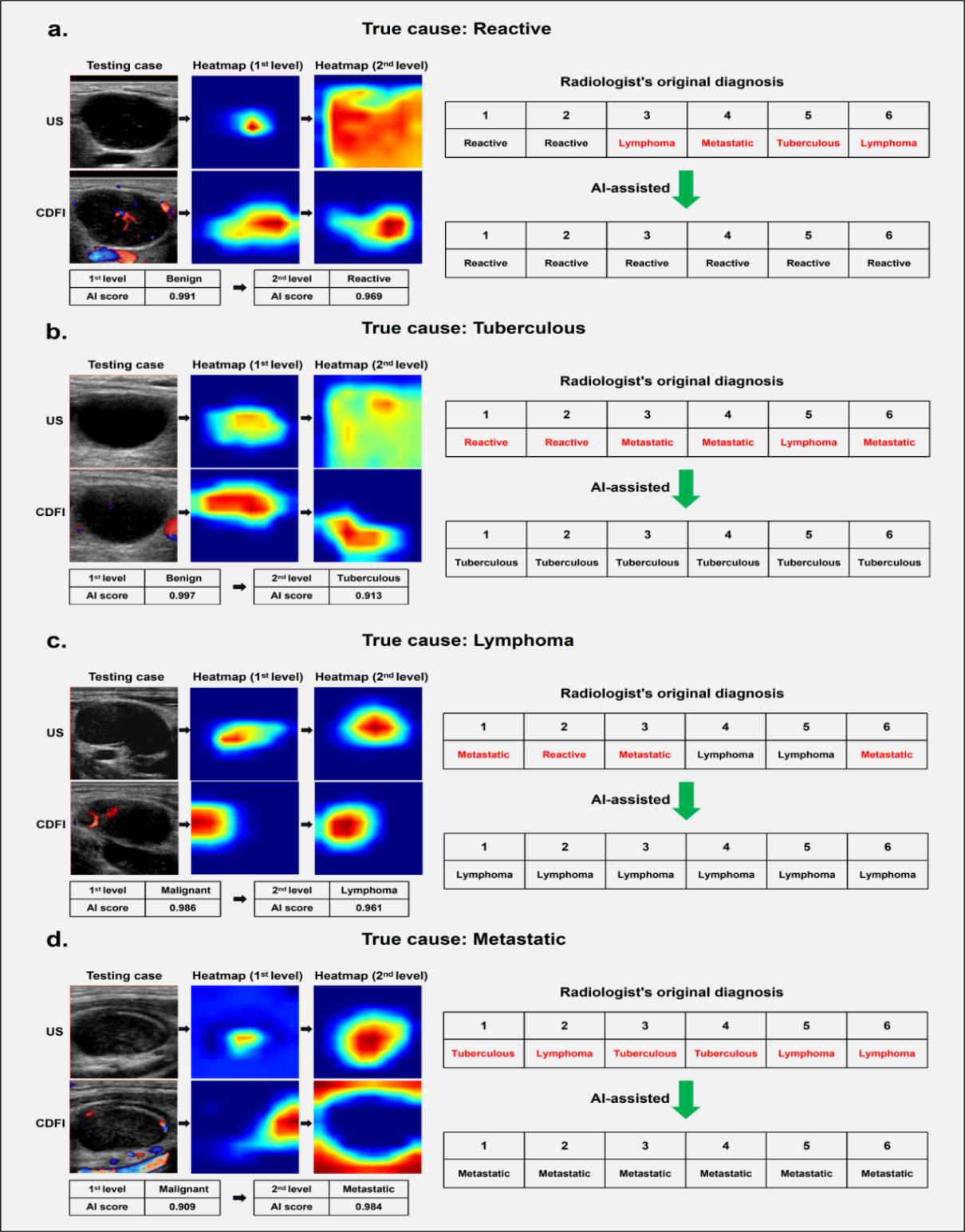


**Fig. S2. Typical cases of CLA-HDM guiding radiologists to make correct decisions.**

a) A case of reactive hyperplasia. With the assistance of CLA-HDM, the accuracy of six radiologists improved from 33.3% to 100%. b) A case of tuberculous lymphadenitis. With the assistance of CLA-HDM, the accuracy of six radiologists improved from 0% to 100%. c) A case of lymphoma. With the assistance of CLA-HDM, the accuracy of six radiologists improved from 33.3% to 100%. d) A case of metastatic carcinoma. With the assistance of CLA-HDM, the accuracy of six radiologists improved from 0% to 100%. In these four cases, the scores of CLA-HDM accurately predicted the etiology of patients with unexplained CLA. Both the differentiation of benign and malignant CLA and the further identification of the four etiologies in the highlighted areas of the heatmaps were consistent with the correspondingly regular pattern of the findings.


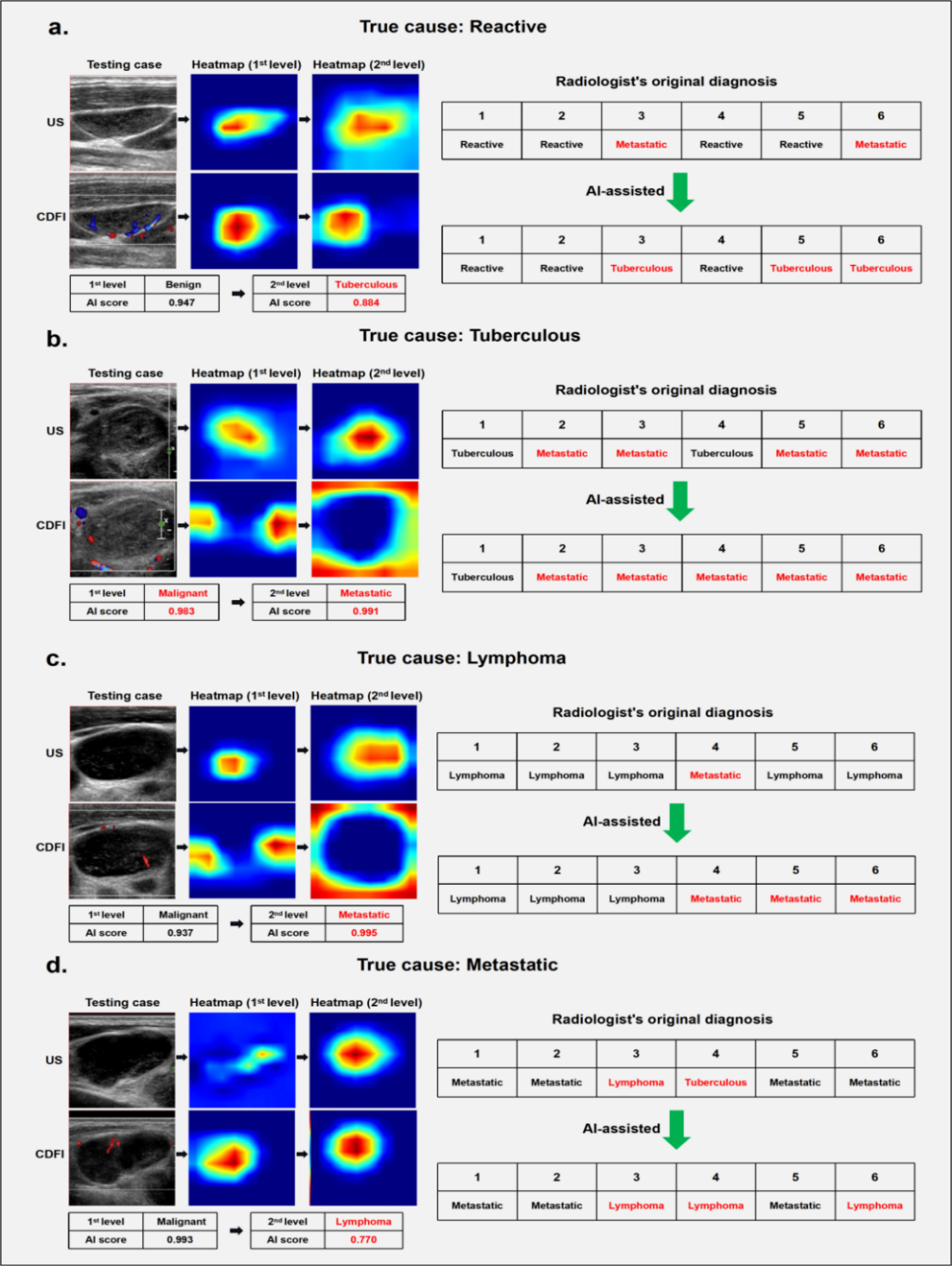


**Fig. S3. Typical cases of CLA-HDM that misled radiologists to make incorrect decisions.**

a) A case of reactive hyperplasia was incorrectly predicted by the model to be tuberculous lymphadenitis. With access to the information from CLA-HDM, the number of radiologists who made the wrong diagnosis increased from 2 to 3. b) A case of tuberculous lymphadenitis was incorrectly predicted by the model to be metastatic carcinoma. With access to the information from CLA-HDM, the number of radiologists who made the wrong diagnosis increased from 4 to 5. c) A case of lymphoma was incorrectly predicted by the model to be metastatic carcinoma. With access to the information from CLA-HDM, the number of radiologists who made the wrong diagnosis increased from 1 to 3. d) A case of metastatic carcinoma was incorrectly predicted by the model to be lymphoma. With access to the information from CLA-HDM, the number of radiologists who made the wrong diagnosis increased from 2 to 3. In these four cases, the radiologists with junior-level experience (radiologists-5 and -6) were most likely to change their original diagnosis, and their errors may have been related to inexperience or carelessness.


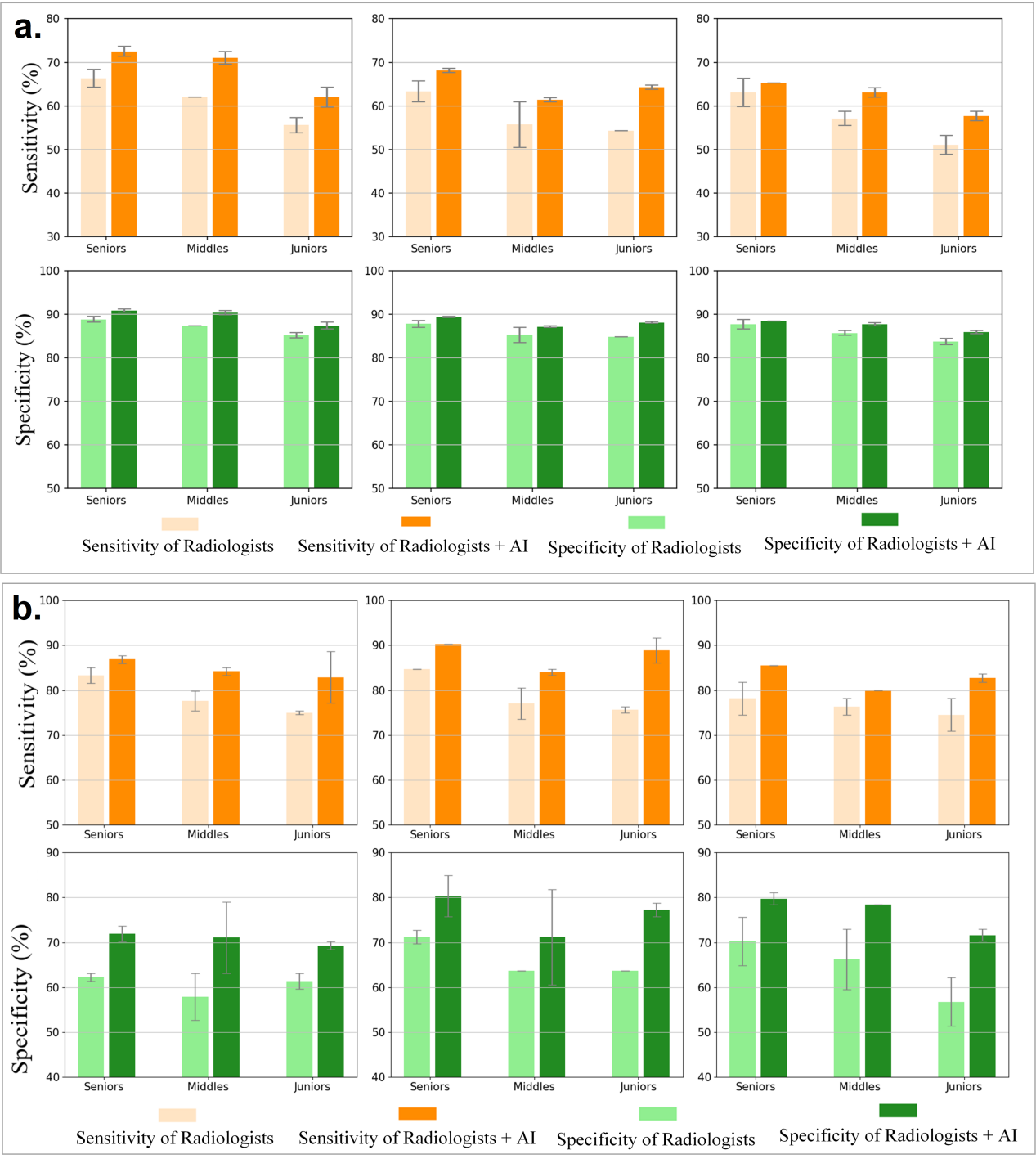


**Fig. S4. Diagnostic performance of different levels of radiologist groups before and after AI-assistance.**

a) Sensitivity and specificity before and after AI assistance for the three different levels of radiologist groups in differentiating four common etiologies of unexplained CLA. b) Sensitivity and specificity before and after AI assistance for the three different levels of radiologist groups in differentiating benign and malignant CLA.
